# STN-DBS exerts neuroprotection and anti-inflammatory effects in a Parkinson’s disease rat model

**DOI:** 10.3389/fimmu.2026.1896631

**Published:** 2026-08-26

**Authors:** Ying Yuan, Qidan Liu, Peng Liu, Lili Guo, Jin Zhang, Jiang Xu, Qiang Liu, Ruxiang Xu, Huan Xiong

**Affiliations:** 1 Department of Neurosurgery, Sichuan Provincial People’s Hospital, School of Medicine, University of Electronic Science and Technology of China, Chengdu, China; 2 Department of Neurology, The First Affiliated Hospital of Guangzhou Medical University, Guangzhou, China

**Keywords:** deep brain stimulation, microglial polarization, neuroinflammation, NF-κB, Parkinson’s disease

## Abstract

**Background:**

Parkinson’s disease (PD) is a progressive neurodegeneration disease characterized by dopaminergic (DA) neuron loss, with chronic neuroinflammation. Subthalamic nucleus deep brain stimulation (STN-DBS) is clinically effective for the relief of parkinsonism motor symptoms. Here, we used a homemade device to investigate the effect of STN-DBS on neuroprotection and chronic neuroinflammation in a unilateral 6-hydroxydopamine (6-OHDA)-induced PD rat model.

**Method:**

Male Sprague–Dawley rats received 6-OHDA injections into the striatum, followed by ipsilateral STN electrode implantation and high-frequency stimulation. Motor function was assessed by open-field and apomorphine-induced rotation tests. DA neuron survival, glial phenotype changes, and nuclear factor (NF)-κB pathway activity in the nigrostriatal system were evaluated using Western blotting, immunofluorescence, and RT-qPCR.

**Results:**

STN-DBS improved motor deficits and decreased the loss of tyrosine hydroxylase-positive neurons. It promoted astrocytes presented a neuroprotective phenotype and increased expression of brain-derived neurotrophic factor, and microglia mainly presented an anti-inflammatory M2 phenotype instead of a pro-inflammatory M1 phenotype. These effects may be associated with IκB-α stabilization, the suppression of NF-κB hyperactivation, and the consequent reduction in the release of downstream pro-inflammatory cytokines.

**Conclusion:**

Our findings highlight that our homemade device for STN-DBS is capable of inhibiting NF-κB, modulating glial phenotypes, mitigating neuroinflammation, and ultimately ameliorating parkinsonism deficits.

## Highlights

- Homemade device for STN-DBS could ameliorate parkinsonism deficits.
- The therapeutic effect of STN-DBS is involved in mitigating neuroinflammation.
- Microglia predominantly presented anti-inflammatory M2 states in the STN-DBS treatment.
- STN-DBS reshapes reactive astrocyte and promotes neurotrophic factor expression.
- The anti-inflammatory effect of STN-DBS is related to NF-κB signaling.

## 1 Introduction

Parkinson’s disease (PD) is the second most common neurodegenerative disease, with the pathological hallmark of progressive loss of dopaminergic (DA) neurons in the substantia nigra (SN) pars compacta and motor manifestations such as resting tremor, bradykinesia, and rigidity (1). With the acceleration of global population aging, both the incidence and prevalence of PD continue to rise, imposing a substantial medical and socioeconomic burden (2–4). Although the precise pathogenesis of PD remains elusive, it is widely accepted that genetic factors, environmental exposures, oxidative stress, mitochondrial dysfunction, and neuroinflammation act in concert to drive disease progression (5).

Neuroinflammation is considered a key contributor to the pathological progression of PD (6). Cerebrospinal fluid and postmortem profiles from patients with PD have revealed widespread microglial activation and elevated levels of pro-inflammatory cytokines (7, 8). Overactivated microglia can release cytotoxic mediators, triggering neurotoxic cascades (9). Furthermore, reactive astrocytes also play pivotal roles in various neurodegeneration and neuroinflammation diseases (10, 11). Increased reactive microglia and upregulated pro-inflammatory cytokines are key characteristics of PD (12, 13), whereas elevated anti-inflammatory factors, such as interleukin-10 (IL-10), may counteract disease progression (14). Therefore, targeting neuroinflammation has emerged as a promising therapeutic strategy for PD.

Deep brain stimulation (DBS) is an established and effective neurosurgical intervention for alleviating motor symptoms in PD (15). Prospective clinical studies have shown that early DBS can reduce the need for complex pharmacological regimens and provide sustained motor benefits (16). Common targets for DBS treatment of PD include subthalamic nucleus (STN) and internal globus pallidus, both of which can improve motor function of PD. However, STN is more effective in reducing the use of DA drugs (17). A 5-year randomized controlled trial further highlighted the long-term efficacy of DBS in STN (STN-DBS) in improving motor dysfunction and enhancing quality of life (18). Collectively, these findings suggest that STN-DBS could produce marked symptomatic relief and confer neuroprotective effects.

Studies have indicated that glia play a role in the therapeutic effects of DBS. For instance, DBS can activate astrocytes, thereby inducing the release of various neurotransmitters, such as glutamate and adenosine, regulating neuronal activity, and promoting the expression of brain-derived neurotrophic factors (BDNFs) (19, 20).

Here, we employed a 6-hydroxydopamine (6-OHDA)-induced PD rat model to systematically evaluate the effects of our homemade device for STN-DBS on motor function and DA neuronal survival, and to explore its regulatory roles in microglial polarization and astrocytic phenotype, neurotrophic factor expression, and the nuclear factor (NF)-κB signaling pathway activation.

## 2 Materials and methods

### 2.1 Animals

All animal studies were performed in accordance with the guidelines of the Animal Care and Use Committee (No. 2021228) of Sichuan Provincial People’s Hospital. Male Sprague–Dawley (SD) rats weighing 240–300 g were purchased from Chengdu Ensiweier Biotechnology Co. Ltd. and housed and bred in a standard SPF animal facility. The SD rats were randomly assigned to solvent or 6-OHDA stereotactic injection, then the rats with solvent injection were randomly assigned to the Control or the Control-DBS group, the rats with 6-OHDA were randomly assigned to the PD or the PD-DBS group only after they were validated as PD models.

### 2.2 Establishment of the PD model

Rats were anesthetized with intraperitoneal injection of a mixed solution containing 6% chloral hydrate and 12% urethane (Sangon Biotech) in 0.9% saline with 10 mL/kg. Anesthetized rats were fixed in a stereotaxic apparatus (RWD) and shaved scalp hair. Then, the 6-OHDA [Sigma-Aldrich, 5 μg/μL, dissolved in 0.9% saline with 0.02% ascorbic acid (AA)] was injected at two points with a rate of 150 nL/min in the left striatum as follows: anteroposterior (AP) +1.6 mm, mediolateral (ML) +2.4 mm, dorsoventral (DV) −4.2 mm and AP −0.2 mm, ML + 2.6 mm, DV −7.0 mm (21); each site received 1.2 μL. The rats in the Control and Control-DBS group received a solvent of 0.9% saline with 0.02% AA. After injection, the needle was left in place for 5 min before being withdrawn slowly.

### 2.3 Behavioral test

#### 2.3.1 Open field test

Rats were placed in a Plexiglas chamber (80 × 80 × 40 cm) to adapt for 5 min and then allowed to freely explore for 20 min. A JLBehv-LAG-1 behavioral analysis system (Beijing KeyueHuacheng Co., Ltd.) was used to record and analyze activity within the 20 min. After each trial, the chamber was cleaned thoroughly with distilled water followed by 75% ethanol to remove residual odors. In the activity analysis, the travel distance and the speed of movement of the rats were all calculated.

#### 2.3.2 Apomorphine-induced rotation test and stimulation-APO test

Three weeks after 6-OHDA injection, rats received a subcutaneous injection of apomorphine (APO) (0.5 mg/kg, Sigma-Aldrich), and then recorded the number of rotation to the contralateral for 1 h. Rats exhibiting more than seven contralateral rotations per minute were considered as successful PD models, and then they were randomly assigned into the PD or PD-DBS groups.

**Figure 1:**
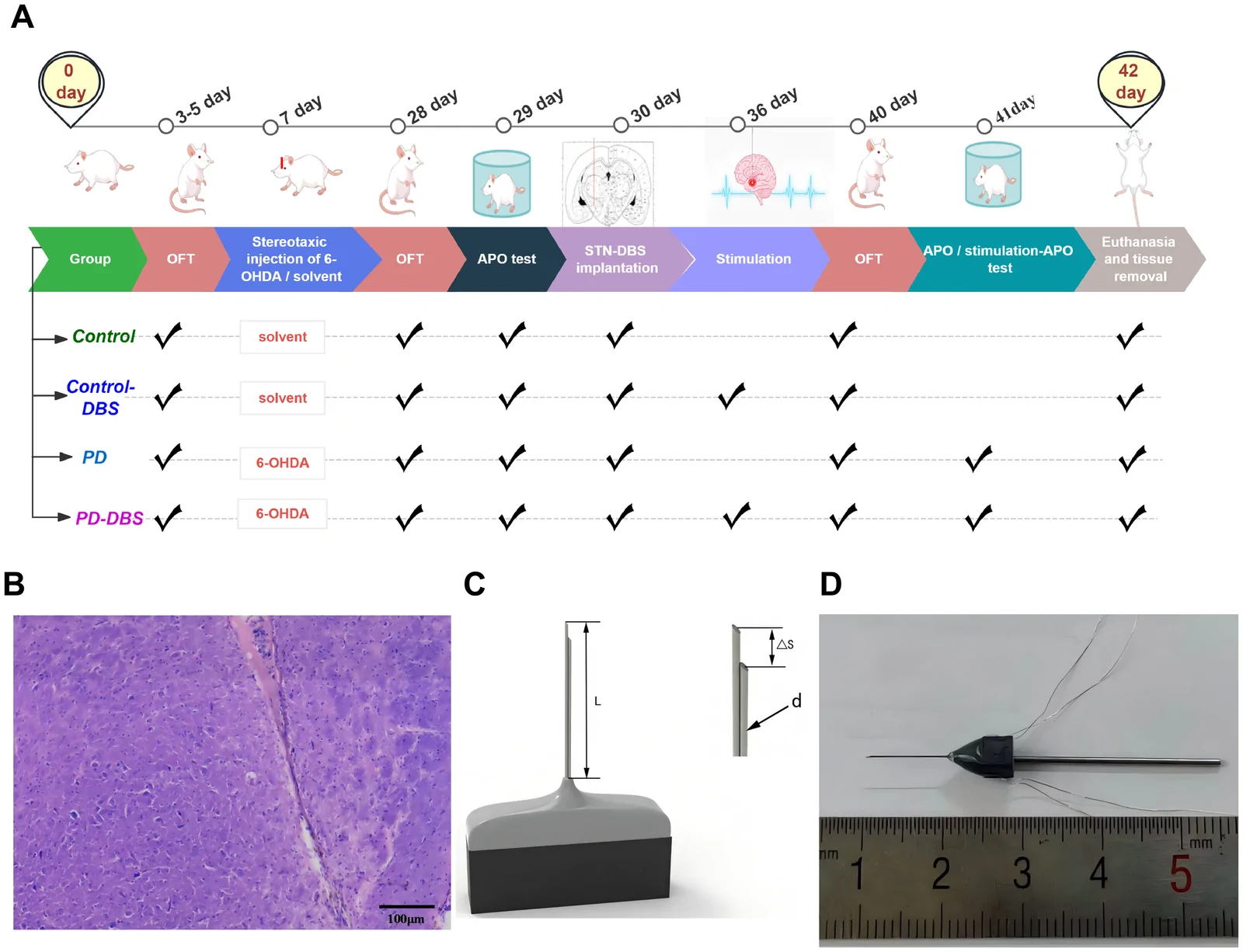
Experimental workflow and verification of the location of implanted electrode. **(A)** Schematic of the experimental workflow. **(B)** HE staining shows the electrode implantation site in the STN. Scale bar = 100 μm. **(C)** Magnified image and parameters of the custom-made electrode tip. *L*, electrode length = 10 mm. Δ*S*, the length difference between the longest and shortest electrode wires = 500 μm. *d*, electrode diameter = 100 μm. **(D)** Physical image of the custom-made electrode. The tiny tip of the electrode on the left would be implanted into the brain, while the swollen part in the middle would be exposed on the surface of the skull and is used to connect to the stimulation output controller during stimulation. The small rod on the right is used to fix the electrode to the stereotaxic instrument during electrode implantation. This rod will be removed after the electrode implantation is completed.

The Stimulation-APO test refers to rats in the PD-DBS groups receiving 15 min of electrical stimulation beginning at 15 min after the subcutaneous injection of APO on day 41, as shown in **Figure 1A**. The number of contralateral rotations per minute was recorded for the 15 min prior to stimulation, the 15 min during stimulation, and the 30 min following the cessation of stimulation separately. The rats in the PD-DBS group will be used for subsequent analysis if they exhibited a significant reduction in contralateral rotation frequency during the stimulation in the Stimulation-APO test. For rats in the PD group, only subcutaneous injection of APO was administered, with no electrical stimulation applied, and then the number of contralateral rotations per minute during the initial 15 min and the last 30 min were recorded.

### 2.4 Electrode implantation and stimulation

In our study, microelectrodes made from nickel–titanium alloy wires were used as the stimulation electrodes, which were connected to the homemade modulator that delivered high-frequency stimulation. The electrode wires were formed by bonding three rigid materials, with a beveled tip to facilitate implantation. The magnified and representative images of the electrode are shown in **Figures 1C, D**, respectively. The electrodes were implanted into the left STN (AP −3.6 mm, ML −2.7 mm, DV −7.8 mm) as previously described (21), and stainless screws were anchored to the skull to secure the ground wires of the electrode. Dental cement was applied to fix the electrode assembly. The electrical pulse parameters used for stimulation were a frequency of 150 Hz, a voltage of 2 V, and a width of 90 μs, which were developed based on a systematic review (22). Rats in both the PD-DBS and Control-DBS groups received 1 h of electrical pulse stimulation on day 36.

### 2.5 Tissue processing

At the end of the experiment, typically on day 42, rats were anesthetized with isoflurane and sacrificed. In each group, the animals were perfused with phosphate-buffered saline (PBS) or PBS followed by 4% paraformaldehyde (PFA, dissolved in 0.1 M PBS, pH 7.4) transcardially based on the downstream assays. The animals used for Western blotting (WB) or RT-qPCR were perfused with PBS and then quickly dissected to harvest striatum and SN and stored at −80 °C. The animals used for staining were perfused with PBS followed by PFA. Then, the brain was removed and postfixed at 4 °C in 4% PFA overnight, followed by cryoprotection in 30% sucrose at 4 °C for 2–3 days. The striatum and SN were cut into 30-μm sections on a cryostat microtome (FS800A, RWD) for histological analysis.

### 2.6 Western blotting

The SN was rinsed in ice-cold PBS and homogenized with RIPA buffer (P0013B, Beyotime) on ice for 30 min. After centrifugation at 13,000 × *g* for 20 min at 4 °C, the supernatants were harvested for analysis. We used a BCA protein assay kit (E112-02-BOX2, Vazyme Biotech) to calculate the protein concentration. An equal amount of protein from each sample was separated by SDS-PAGE and transferred onto PVDF membrane (Millipore), as previously described (23). After blocking with 5% skim milk for 1 h at room temperature, the membranes were incubated overnight with primary antibodies (**Supplementary Table 1**), and then incubated for 2 h at room temperature with IgG-HRP conjugated antibodies (**Supplementary Table 1**). The immunoreactive signals were visualized with an enhanced chemiluminescence detection system (P0018AM, Beyotime) and Tanon-5200 system and then quantified with using ImageJ (1.49m, NIH) software. For quantification of protein levels in the SN, it was normalized to GAPDH ipsilateral. It should be noted that when analyzing TH expression, we calculated the ratio of TH levels on the electrode implantation side to the contralateral side. For other proteins, we only analyzed the expression levels on the electrode implantation side.

### 2.7 Immunofluorescence and hematoxylin and eosin staining

For immunofluorescence, the sections were washed in PBST (0.1% Tween-20 in PBS), permeabilized by incubation in 0.5% Triton X-100 in PBS for 20 min at room temperature, blocked by incubation in blocking solution (P0260, Beyotime) for 30 min, and then incubated overnight at 4 °C with primary antibodies (**Supplementary Table 1**) followed by Alexa Fluor™-conjugated secondary antibodies for 2 h at room temperature. Slices were incubated with DAPI-containing antifade medium (P0131, Beyotime), covered with coverslips, and then imaged using a fluorescence microscope (VS200, Olympus).

For hematoxylin and eosin (HE) staining, the sections were immersed in hematoxylin solution for 3 min and subsequently rinsed with distilled water to remove excess solution. After that, the sections were dehydrated in 95% ethanol for 1 min and then stained in eosin solution for 15 s. Last, the stained sections were dehydrated in graded ethanol, cleared in xylene, and mounted with neutral resin. All steps were performed in a fume hood to ensure experimental safety. Thereafter, these sections were observed under a light microscope (SWE-CX63, Servicebio) to evaluate the electrode location in the STN.

After that, the slices were quantified using ImageJ (1.49m, NIH) software.

### 2.8 RT-qPCR

The mRNA levels of IL-1β, IL-10, and tumor necrosis factor-α (TNF-α) were quantified by RT-qPCR. The total RNAs were extracted from left SN tissue using TRIzol reagent (G3013, Servicebio) according to the manufacturer’s instructions. Then, the lysates were precipitated with isopropanol, washed with 75% ethanol, dried, and dissolved in RNase-free water. Total RNA was reverse transcribed into cDNA using a SweScript All-in-One First-Strand cDNA Synthesis SuperMix for qPCR (G3337, Servicebio). qPCR was performed in triplicate for each sample with gene-specific primers (**Supplementary Table 2**) using the QuantStudio PCR system. The GAPDH gene served as an internal control, and target gene expression was calculated relative to the GAPDH using the formula 2^−ΔΔCT^.

### 2.9 Statistical analysis

Data are presented as mean ± standard error of the mean (SEM) and analyzed with GraphPad Prism 9.0 statistical software. Comparison between two groups was assessed using Student’s *t*-test, while multiple-group comparisons were performed using one-way analysis of variance (ANOVA) or two-way ANOVA followed by Tukey’s *post hoc* multiple comparison test. *p* < 0.05 was considered statistically significant.

## 3 Results

### 3.1 Experimental scheme and verification of electrode placement

The experimental workflow for PD model establishment, phenotype detection, and STN electrical stimulation is illustrated in **Figure 1A**. Briefly, rats first underwent a baseline open field test (OFT), followed by stereotaxic injection of either vehicle or 6-OHDA into the striatum. Three weeks after lesion induction, a second OFT was performed, followed by an APO-induced rotation test to confirm successful establishment of the PD model. Subsequently, electrodes were implanted into the STN in all four groups, and electrical stimulation was applied to the Control-DBS and PD-DBS groups. A third OFT was then conducted in all rats, followed by a Stimulation-APO test in the PD and PD-DBS groups. Finally, the rats were all sacrificed, and brain tissue was harvested for subsequent analysis. The electrode trajectories and tips within the left STN were verified using HE staining (**Figure 1B**).

### 3.2 STN-DBS significantly alleviates motor deficits in 6-OHDA-induced PD rats

**Figure 2:**
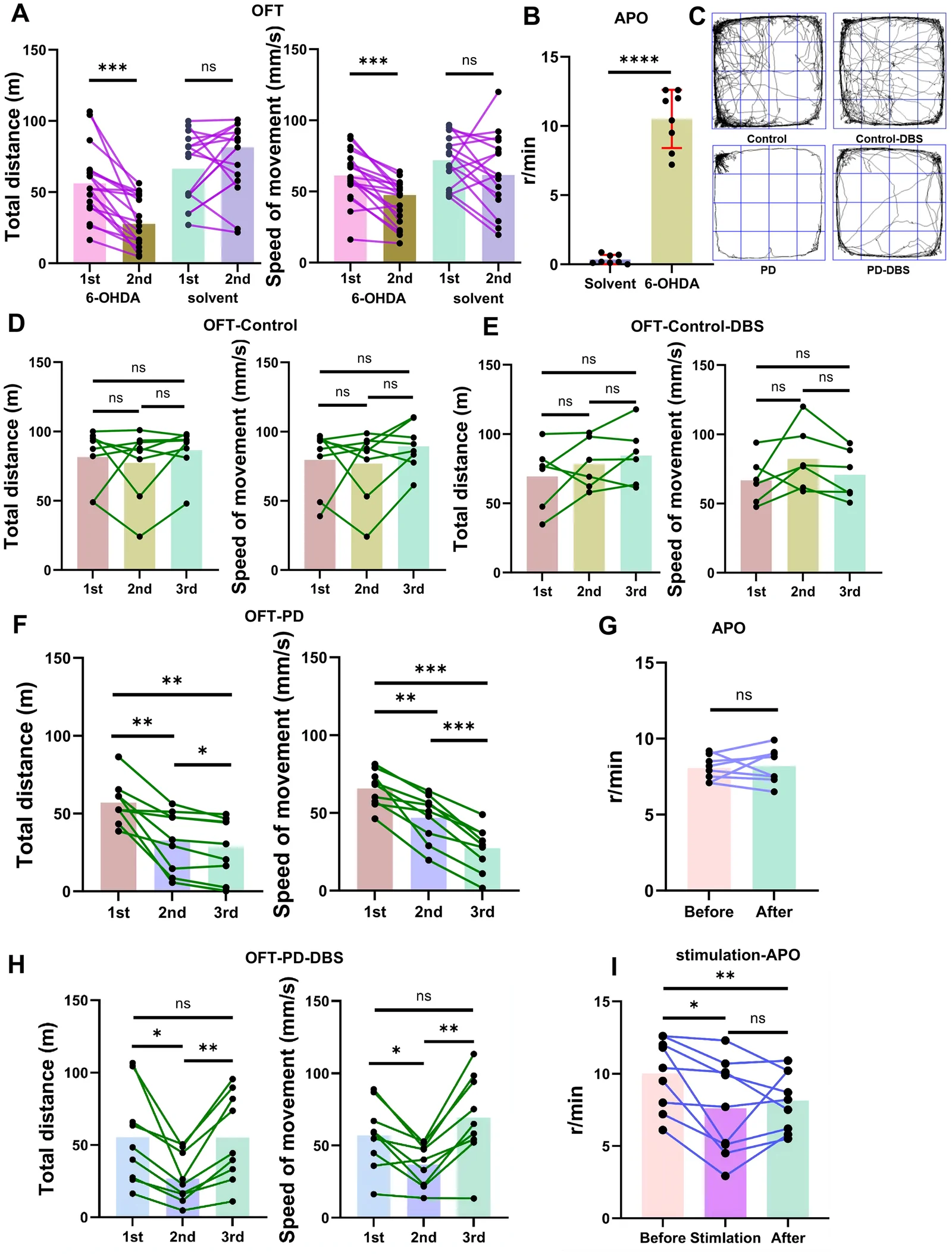
STN-DBS ameliorated motor dysfunction in the 6-OHDA-induced PD rat model. **(A)** Total distance traveled and mean movement speed of rats in the first and second OFT before and after 6-OHDA or solvent treatment. **(B)** APO-induced rotations in rats after solvent or 6-OHDA injection. r/min represent number of rotations per minute. **(C)** Representative movement traces of rats in each group in the third OFT. **(D)** Total distance traveled and mean movement speed in the Control group in the three OFTs. **(E)** Total distance traveled and mean movement speed in the Control-DBS group in the three OFTs. **(F)** Total distance traveled and mean movement speed in the PD group in the three OFTs. **(G)** The second APO-induced rotation test in the PD group. The number of contralateral rotations per minute during the initial 15 min and the last 30 min were defined as before and after. **(H)** Total distance traveled and mean movement speed in the PD-DBS group in the three OFTs. **(I)** Rotation detected in the PD-DBS group rats within the Stimulation-APO test. The number of contralateral rotations per minute during 15 min prior to stimulation, the 15 min during stimulation, and the 30 min following the cessation of stimulation were defined as before, stimulation, and after. Each point on the graph represents a sample; the points before and after the line correspond to the sample. ns, not significant; *p < 0.05, **p < 0.01, ***p < 0.001, ****p < 0.0001.

To evaluate both the validity of the PD model and the therapeutic efficacy of STN-DBS, we conducted the OFT and the APO-induced rotation test. In the OFT, rats exhibited a marked reduction in total distance traveled and average speed after 6-OHDA induction, whereas there was no difference after solvent stereotaxic injection (**Figure 2A**). In the APO-induced rotation test, 6-OHDA-lesioned rats exhibited strong contralateral rotation (**Figure 2B**), confirming the severe unilateral damage to the SN–striatum pathway. These results indicated the successful construction of the PD model.

The movement traces of rats from the four groups in the third OFT showed that STN-DBS could alleviate motor dysfunction (**Figure 2C**). Then, we calculate the total distance traveled and speed of movement of the rats in all of the three OFTs and found that the electrode implantation did not affect the total distance traveled and speed of movement in the Control group rats (**Figure 2D**), indicating that the electrode implantation in the STN was tolerable. Comparison before and after the electrical stimulation in the STN revealed that the total distance traveled and the speed of movement in the Control-DBS group did not change, but significantly increased in the PD-DBS group (**Figures 2E, H**), indicating that the electric stimulation in the STN of control rats was tolerable, and the STN-DBS could alleviate the motor dysfunction of PD. Notably, in PD group rats, the motor dysfunction continued to worsen, represented as a continued decrease in total distance traveled and speed of movement compared to that detected before electrode implantation (**Figure 2F**).

In addition, in the Stimulation-APO test on PD-DBS rats and second APO test on PD rats, our results showed that stimulation in the STN can reduce the number of APO-induced rotations in PD-DBS rats (**Figures 2G, I**). Together, these results demonstrate that STN-DBS effectively mitigates motor impairments in the 6-OHDA-induced rat model of PD.

### 3.3 STN-DBS rescues DA neurons and suppresses pathological α-synuclein aggregation

To further clarify the establishment of the PD rat model and the neuroprotective effects of DBS, we performed immunofluorescence staining on brain sections to analyze the expression of TH in SN and striatum, and of alpha-synuclein (α-syn) in SN.

**Figure 3:**
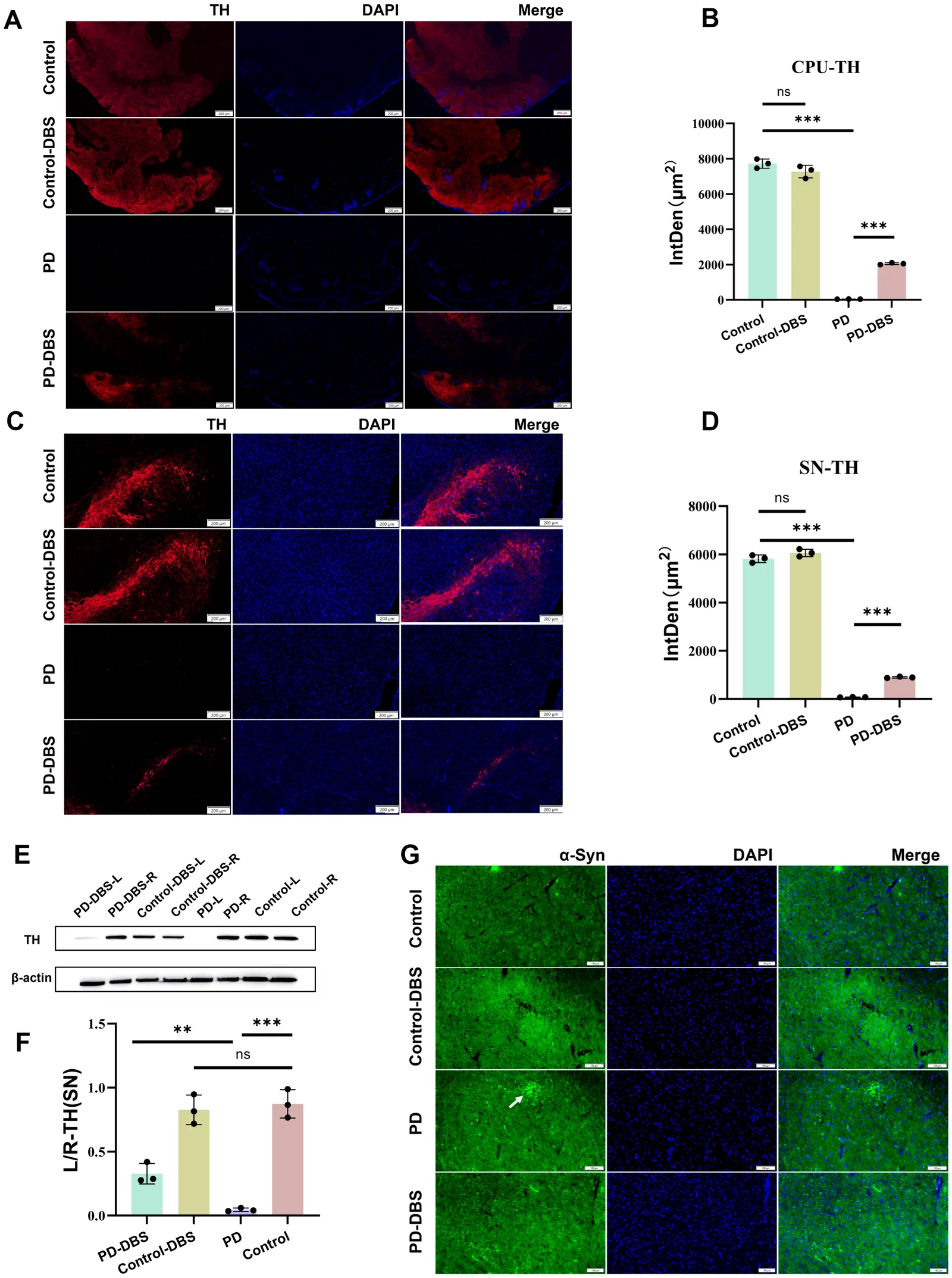
DBS preserves DA neurons and reduces α-synuclein pathology in a PD rat model. **(A)** Images of TH-positive fibers in the left striatum (CPU) of each group. Scale bars = 200 μm. **(B)** Statistical histogram of fluorescence intensity in the left CPU of each group. **(C)** Images of TH-positive fibers in the left substantia nigra (SN) of each group. Scale bars = 200 μm. **(D)** Statistical histogram of fluorescence intensity in the left SN of each group. **(E, F)** Immunoblotting and quantification of TH protein levels in the SN of brain tissues isolated from each group. The protein expression was normalized to GAPDH. The histogram showed the ratio of the left SN to the right SN. **(G)** Images of α-synuclein immunofluorescence staining in the left SN. The arrow indicates where α-syn is located. Scale bar = 100 μm. Data are means ± SEM. Each point on the graph represents a sample. ns, not significant. *p < 0.05, **p < 0.01, ***p < 0.001.

In the left striatum, the PD group exhibited a decrease in TH-positive fiber density compared with the Control group, whereas STN-DBS markedly increased TH expression in the PD-DBS group compared with the PD group (**Figures 3A, B**). In the Control-DBS group, stimulation did not alter TH expression in the striatum, indicating that DBS in the SN was tolerable.

In the SN, there was a pronounced loss of TH-positive neuronal density in the PD group compared to the Control group, which is higher in the PD-DBS group than in the PD group, indicating that STN-DBS intervention effectively attenuated the TH loss (**Figures 3C, D**), while there was no difference between the Control-DBS and Control group, indicating that DBS in the SN was tolerable (**Figures 3C, D**). Our WB results confirmed that TH expression in the SN was markedly reduced in the PD group compared to the Control group, while it was increased in the PD-DBS group compared to the PD group, and has no difference between the Control and the Control-DBS group (**Figures 3E, F**).

Abnormal α-syn aggregation was a hallmark of PD pathology (24); we then detected the expression of α-syn in the SN. In the PD group, the pathological aggregation of α-syn was obvious, while in the PD-DBS group, the α-syn distribution more closely resembled the diffuse pattern observed in control animals (**Figure 3G**). Collectively, these results indicate that STN-DBS not only protects DA neurons from degeneration but also mitigates α-syn aggregation.

### 3.4 STN-DBS prevents microglial over-activation and promotes an anti-inflammatory phenotype

Considering the important role of neuroinflammation in PD, we then investigated the effects of STN-DBS on microglia in the SN, especially focused on the microglial activation, polarization phenotypes, and the expression of related inflammatory factors.

**Figure 4:**
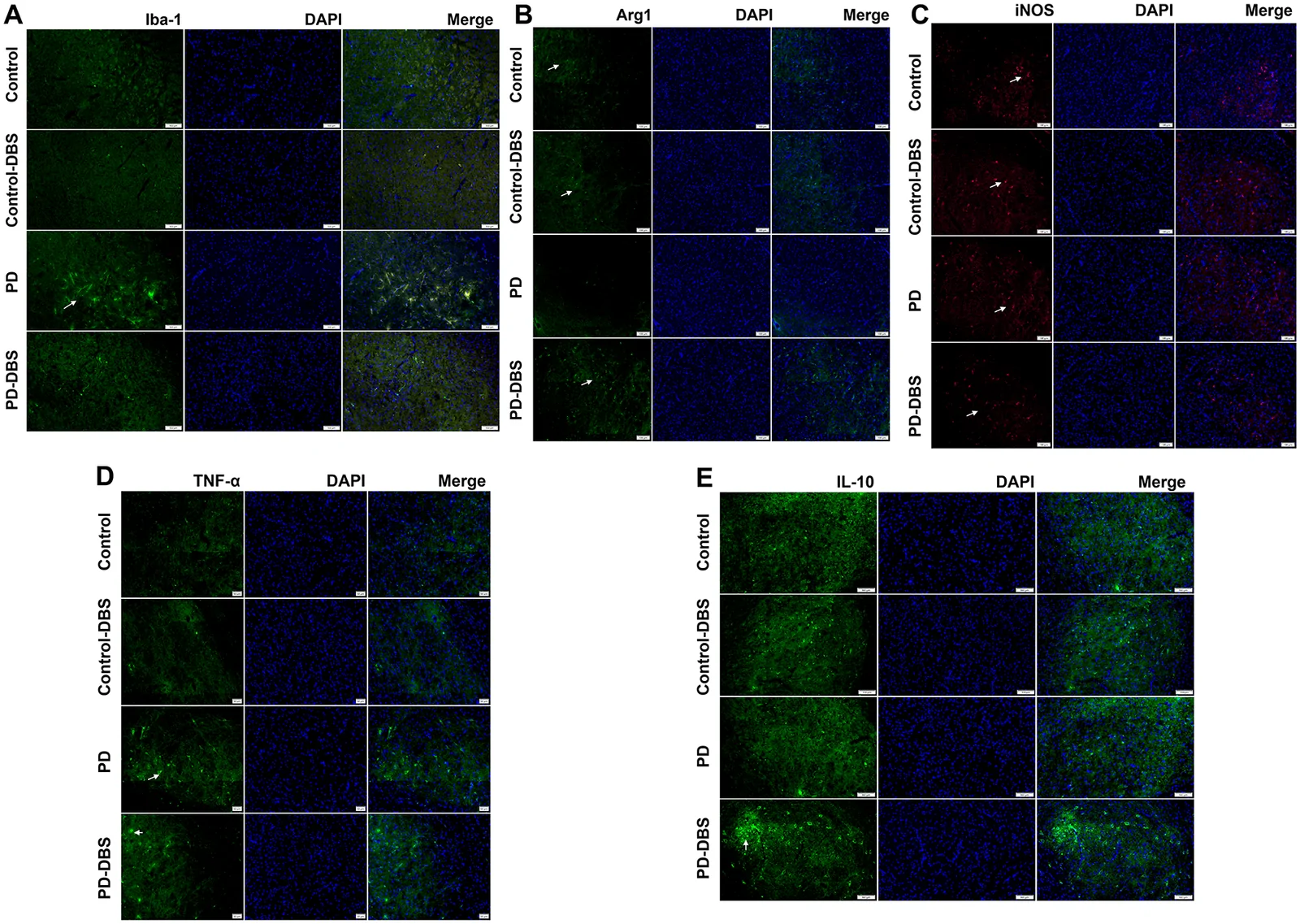
DBS affects microglial expression and their phenotype in the SN of PD model rats. **(A)** Images of Iba-1 staining in the left SN across groups. White arrows indicate a typical Iba-1-positive fluorescent signal. Scale bar = 100 μm. **(B, C)** Images of iNOS **(C)** and Arg1 **(B)** immunostaining in the left SN of each group. White arrows indicate a typical iNOS-positive signal and a typical Arg1-positive signal, respectively. Scale bar = 100 μm. **(D)** Images of TNF-α immunostaining in the left SN of each group. White arrows indicate a typical TNF-α-positive signal. Scale bar = 50 μm. **(E)** Images of IL-10 immunostaining in the left SN of each group. White arrows indicate a typical IL-10-positive signal. Scale bar = 100 μm.

**Figure 4A** shows that ionized calcium binding adapter molecule 1 (Iba-1) positive microglia were markedly increased in the PD group. In the PD-DBS group, the STN-DBS markedly suppressed Iba-1 expression, indicating that STN-DBS can effectively alleviate Iba-1-positive microglia higher expression in SN under PD pathology.

Inducible nitric oxide synthase (iNOS) is widely recognized as a hallmark of M1 type with pro-inflammatory action, while arginase 1 (Arg1) is recognized as a hallmark of M2 type with anti-inflammatory action (25). In our results, the iNOS expression in the PD group was higher than that in the other three groups, while Arg1 expression was scarcely detected in the PD group but obvious in the other three groups (**Figures 4B, C**), indicating that STN-DBS could improve the increased M1-type and decreased M2-type microglia in the PD rats. Consistent with this phenotypic change, STN-DBS significantly ameliorates the increased expression of the pro-inflammatory factor TNF-α and decreased expression of the anti-inflammatory factor IL-10 in the PD group (**Figures 4D, E**), thereby establishing a neuroprotective inflammatory microenvironment. In general, STN-DBS could inhibit the pro-inflammatory response of microglia while promoting the anti-inflammatory M2 phenotype expression.

### 3.5 STN-DBS reshapes reactive astrocyte and promotes neurotrophic factor expression

To explore the effects of STN-DBS on astrocytes, we assessed the expression of glial fibrillary acidic protein (GFAP) and BDNF.

**Figure 5:**
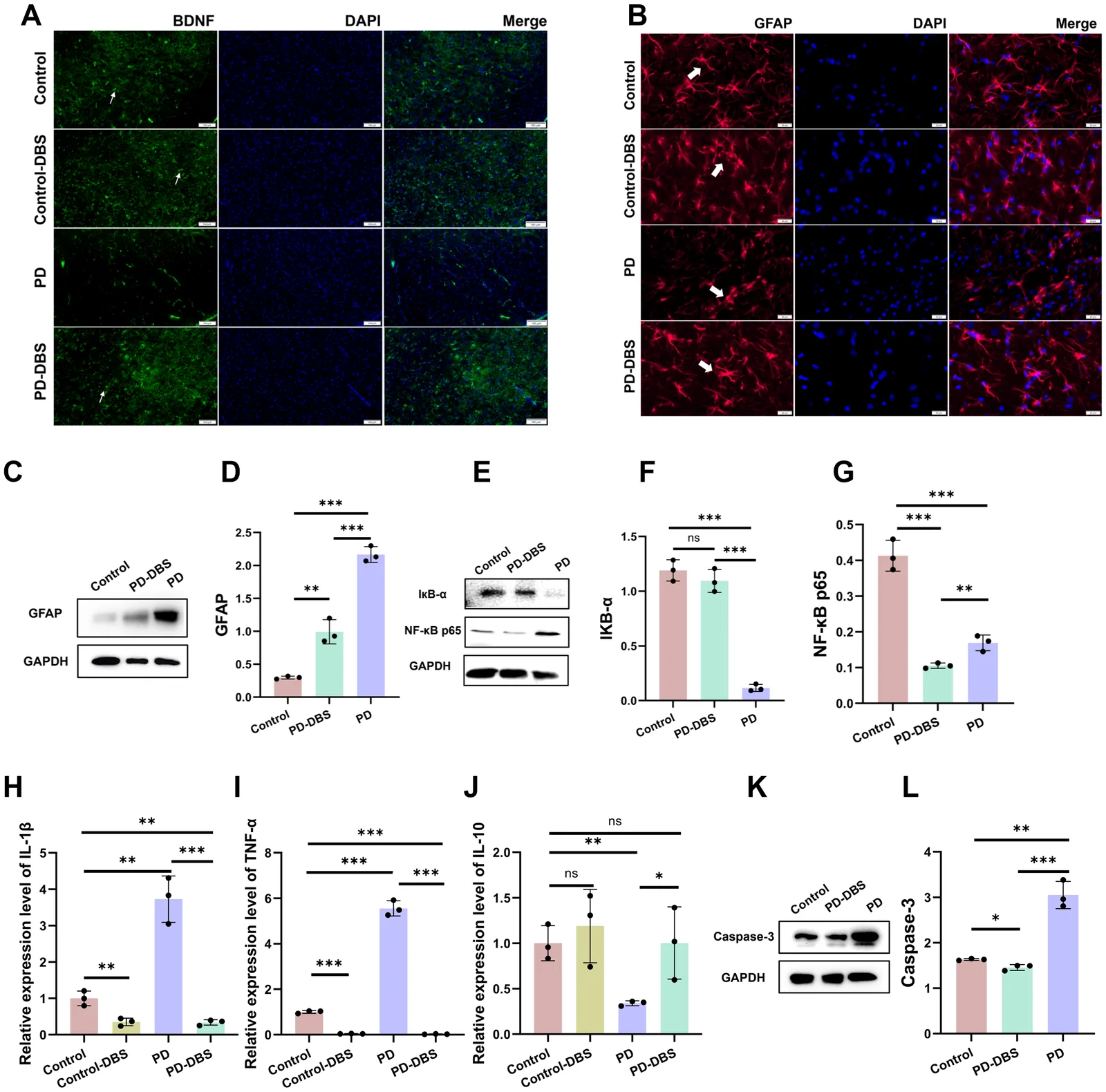
STN-DBS could reshape reactive astrocyte and modulates NF-κB signaling and apoptosis in the left SN. **(A)** Immunofluorescence staining of BDNF in the left SN. Scale bar = 100 μm. **(B)** Magnified images of GFAP immunofluorescence staining in each group. White arrows indicate a typical structure of GFAP-positive astrocyte. Scale bar = 20 μm. **(C, D)** Immunoblotting and quantification of GFAP protein levels in the left SN of each group. **(E–G)** Immunoblotting and quantification of IκB-α and NF-κB p65 protein levels in the left SN of each group. **(H–J)** Quantification of TNF-α, IL-1β, and IL-10 in the left SN detected with RT-qPCR. **(K, L)** Immunoblotting and quantification of Caspase-3 protein levels in the left SN of each group. Data are means ± SEM. Each point on the graph represents a sample. ns, not significant. *p < 0.05, **p < 0.01, ***p < 0.001.

Immunofluorescence staining showed that astrocytes in the PD group are activated as reactive astrocytes and exhibited hypertrophic cell bodies, and thickened and shortened processes, accompanied by significant depletion of BDNF expression. STN-DBS treatment could alleviate the reactive astrocyte and increase BDNF expression (**Figures 5A, B**). WB indicated a significant increase in GFAP expression in the PD group compared to the Control group, while the GFAP expression decreased in the PD-DBS group compared to the PD group (**Figures 5C, D**), suggesting that STN-DBS could rescue the reactive astrocyte proliferation. Our results showed that STN-DBS could reshape astrocyte function, restores BDNF expression, and effectively alleviates neuroinflammation in the pathological state of PD.

### 3.6 The anti-inflammatory effect of STN-DBS is related to NF-κB signaling

The pro-inflammatory cytokine production in microglia would be mediated by NF-κB signaling (26). To explore whether the effect of STN-DBS on microglia is related to NF-κB signaling, we then examined the expression of the key protein in NF-κB signaling.

In the resting state, NF-κB is sequestered in the cytoplasm by its inhibitor IκB-α, while in the inflammatory stimulating state, IκB-α was phosphorylated and degraded, then NF-κB is activated and released to the nucleus and drives transcription of pro-inflammatory genes (27). NF-κB p65 is the most common and transcriptionally active subunit in the classic NF-κB pathway (28). Our results showed a significant decrease of IκB-α expression and an increase of NF-κB p65 in the PD group, whereas STN-DBS treatment restored the abnormal IκB-α and NF-κB p65 expression (**Figures 5E–G**), suggesting that STN-DBS may block the activation of NF-κB signaling.

We then detected the content of the inflammatory cytokine in SN with RT-qPCR. STN-DBS significantly helped increase the gene expression of pro-inflammatory TNF-α and IL-1β, and decrease the expression of the anti-inflammatory factor IL-10 in PD rats (**Figures 5H–J**).

Extensive stimulation by pro-inflammatory cytokine may induce cell apoptosis; then, we detected the expression of caspase-3 to represent the degree of apoptosis. Our results showed that caspase-3 expression was markedly elevated in the PD group, while it significantly decreased in the PD-DBS group compared to that in the PD group, suggesting the anti-apoptosis effects of DBS (**Figures 5K, L**).

These results indicate the anti-inflammatory effects of STN-DBS and that was associated with the activation of the NF-κB signaling pathway.

## 4 Discussion

This study demonstrated that our homemade device for STN-DBS could improve motor function in the 6-OHDA-induced PD rat model, which is carried out by suppressing neuroinflammation and reshaping glial cell function, suggesting that STN-DBS may intervene in inflammatory pathological processes of PD.

In line with previous studies suggesting that DBS has disease-modifying potential (29–31), our results further confirm the efficacy of our homemade device for STN-DBS in improving motor deficits and rescuing DA neurons, as well as inhibiting the pathological aggregation of α-syn. Given that α-syn aggregates are the core pathological proteins driving neurotoxicity and the spread of inflammation (32–34), our finding provides pathological evidence for the pathologic-modifying potential of DBS.

Our study also suggests that the neuroprotective effects of our homemade DBS may be involved in regulating the neuroinflammatory microenvironment. The phenotypic plasticity of microglia is regarded as a highly appealing therapeutic target in neurodegenerative diseases (35–37). Our study showed that STN-DBS could suppress the excessive expression of the microglia marker Iba-1, suppress the polarization of microglia toward a pro-inflammatory M1 phenotype, and promote the manifestation of the anti-inflammatory M2 phenotype, and identified STN-DBS as a modulator of this process.

An increase in GFAP is typically regarded as an inhibitory glial scar formed after neural injury (38). Our study showed that STN-DBS could suppress the expression of GFAP, which may help to inhibit the formation of glial scar in PD. On the other side, STN-DBS could restore the expression of BDNF in reactive astrocytes, thereby providing nutritional support to damaged neurons. This is consistent with the A1/A2 astrocyte classification concept proposed in recent years (39), suggesting that DBS may present a neurotherapeutic effect by suppressing the presence of astrocyte on an A1-like neurotoxic phenotype while promoting the presence of an A2-like reparative phenotype (40). These revealed the effect of our STN-DBS on astrocytes.

We further demonstrated that STN-DBS inhibits the activation of the NF-κB signaling pathway, thereby reducing the transcription of NF-κB signaling-driven pro-inflammatory genes (TNF-α and IL-1β) and enhancing the transcription of the anti-inflammatory factor IL-10 gene, which then reduces inflammation-induced apoptosis.

Combining these results, it can be inferred that the therapeutic effects of STN-DBS are involved in suppressing the release of inflammatory factors, reshaping glial cell function, and blocking NF-κB signaling activation. These collectively promote the improvement of motor impairment in PD rats.

This study also has certain limitations. The 6-OHDA model mainly simulates acute death of DA neurons and does not fully reproduce the slow-spread α-syn pathology characteristic of human PD. Future studies should validate these findings in genetic or protein-aggregation models that more closely resemble human pathology. Additionally, further confirmation of the causal relationship of the NF-κB pathway using pharmacological or genetic tools or single-cell sequencing technology (41) will be an important direction for future research.

## 5 Conclusion

These findings confirm the neuroprotective effect of our homemade device for STN-DBS on PD, which is involved in modulating glial cell phenotypes, suppressing pro-inflammatory responses and enhancing neurotrophic support, and inhibiting the NF-κB signaling pathway, demonstrating significant potential to slow neurodegenerative pathology. Our results also contribute to the deeper understanding of the therapeutic effect of DBS and explicit molecular targets for therapy of PD with STN-DBS.

## Data Availability

The original contributions presented in the study are included in the article/**Supplementary Material**. Further inquiries can be directed to the corresponding authors.
