# Supplementary material for: STN-DBS exerts neuroprotection and anti-inflammatory effects in a Parkinson’s disease rat model
Source: Front Immunol. 2026 Aug 26;17:1896631. doi: 10.3389/fimmu.2026.1896631 (PMC13553276; doi:10.3389/fimmu.2026.1896631)
Supplement: Supplementary file 1 [file Table1.docx]

Table 1 Antibodies used in this study.

| **Antibodies** | **Supplier** | **Catalogue number** |
| --- | --- | --- |
| BDNF Mouse McAb | proteintech | 66292-1-1g |
| GFAP Polyclonal antibody | proteintech | 16825-1-AP |
| IκBα (L35A5) Mouse mAb (Amino-terminal Antigen) | Cell signaling technology | 4814S |
| Purified anti-α-Synuclein Phospho (Ser129) Antibody | bidegend | 825701 |
| IL-10 Monoclonal antibody | proteintech | 60269-1-lg |
| TNF-α (D2D4) XP Rabbit mAb | proteintech | 11948S |
| iNOS Monoclonal Antibody (CXNFT), eBioscience™ | invitrogen | 14-5920-82 |
| Arginase 1 Polyclonal Antibody | invitrogen | PA585267 |
| Anti-Tyrosine Hydroxylase antibody [EP1533Y] | abcam | ab75875 |
| IBA1 Monoclonal Antibody (GT10312) | invitrogen | MA527726 |
| Anti-NF-kB p65 (phospho S536) antibody [EP2294Y] | abcam | Ab76302 |
| Donkey anti-Rabbit IgG (H+L) Highly Cross-Adsorbed Secondary Antibody, Alexa Fluor™ 647 | invitrogen | 2420695 |
| Goat anti-Rabbit IgG (H+L) Cross-Adsorbed Secondary Antibody, Alexa Fluor™ 594 | invitrogen | 2433881 |
| Goat anti-Mouse IgG (H+L) Cross-Adsorbed Secondary Antibody, Alexa Fluor™ 488 | invitrogen | 2465113 |

Table 2 Gene-specific primers in this study.

| **Primer name** | **primer sequence (5'-3')** |
| --- | --- |
| R-GAPDH-S | CTGGAGAAACCTGCCAAGTATG |
| R-GAPDH-A | GGTGGAAGAATGGGAGTTGCT |
| R-TNF-α-S | CCAGGTTCTCTTCAAGGGACAA |
| R-TNF-α-A | GGTATGAAATGGCAAATCGGCT |
| R-IL1β-S | ACTTGGGCTGTCCAGATGAGAG |
| R-IL1β-A | CGAGTCACAGAGGACGGGCT |
| R-IL10-S | AGGACTTTAAGGGTTACTTGGGT |
| R-IL10-A | ATCATTCTTCACCTGCTCCACT |

R rat. -S sense primer, upstream primer identifier. -A antisense primer, downstream primer identifier.
